# The high-frequency magnitude (**m**_3Hz_) of Italian earthquakes from intensity data – the ICEM Catalog

**DOI:** 10.1038/s41597-026-07523-6

**Published:** 2026-06-06

**Authors:** Stefano Parolai, Daniele Spallarossa, Matteo Picozzi, Rouhollah Amiri Fard, Adrien Oth, Dino Bindi, Andrea Rovida

**Affiliations:** 1 University of Trieste, Trieste, Italy; 2 University of Genoa, Genoa, Italy; 3 National Institute of Oceanography and applied geophysics- OGS, Sgonico, Italy; 4 University of Naples Federico II, Naples, Italy; 5 European Center for Geodynamics and Seismology, Walferdange, Luxembourg; 6 GFZ Helmholtz Centre for Geosciences, Potsdam, Germany; 7 Istituto Nazionale di Geofisica e Vulcanologia, Milan, Italy

## Abstract

The assessment of seismic hazard and risk requires a catalogue of earthquakes (both historical and recorded during the instrumental era), where the location and magnitude of events affecting the study area are reported. Mw is commonly used for this aim, but it is a static measure of earthquake size and it cannot capture the full extent of earthquake source complexity. Other magnitude scales connected to the rupture kinematics and dynamics should complement Mw in hazard studies, but this required efforts in re-analyzing catalogs of intensity data. To this aim, we developed a high-frequency magnitude (**m**_3Hz_) estimated using a random or log-averaged horizontal component of ground motion from frequencies above the highest corner frequency of the earthquake source spectrum. Here, we apply the novel procedure to the intensity data from the Parametric Catalogue of Italian Earthquakes (CPTI15). We derive **m**_3Hz_ for 1,951 earthquakes (from 1117 to 2020). The new earthquake catalog is defined by the acronym ICEM. For all earthquakes a quality index based on the variance-to-mean ratio (VMR) has been assigned.

## Background & Summary

The assessment of seismic hazard and risk requires a catalog of earthquakes (both historical and recorded in the instrumental era) that allows for the estimation of the location and magnitude of events that have affected the study area^1^. The catalog not only allows for the calculation of the necessary statistical parameters regarding the annual rate of occurrence of events and their relative frequency, but also provides the opportunity, for those recorded by seismometric/accelerometric stations, to calculate the ground motion models (GMM) that are necessary for estimating the probability of exceeding different intensity measures (e.g., peak ground acceleration-PGA-, spectral acceleration at different periods-SA) at each site of interest. To this aim, since its introduction, the moment magnitude Mw^2^ has been used as the main magnitude scale for the compilation of seismic catalogs. This magnitude, being linked to the average static displacement of the rupture and its slip area, is not affected by the saturation phenomena typical of other magnitudes^3^, and therefore represents an important measure of earthquake size. However, recent works^4,5^ have demonstrated that Mw is incapable of capturing all characteristics associated with earthquake ruptures. In particular, Mw may not be an adequate descriptor of radiated energy in the frequency range of interest for most building structures.

In order to account for high-frequency radiation in earthquake size estimation, a high-frequency magnitude, **m**, was introduced^6^, which is estimated using a random or logarithmically averaged horizontal component of ground motion from frequencies above the highest corner frequency of the earthquake source spectrum. Furthermore, a relationship between **m** and Modified Mercalli Intensities was proposed^6^, providing a link between engineering ground motion parameters and macroseismic intensity data. This makes **m** useful also for pre-instrumental earthquakes. However, given that the estimation of **m** is based on the high-frequency level of the Fourier spectrum of acceleration, as originally formulated, it is highly sensitive to site effects, which can result in significant discrepancies in the estimation values. Indeed, regarding the magnitude definition, an average spectral amplification of 3 in the frequency band under consideration results in an increase in magnitude of 1.

A recent study^7^ proposed a methodology for estimating the size of an earthquake, whether at local or regional distances, by utilizing the spectral amplitude of acceleration at 3 Hz (**m**_3Hz_). This methodology involves the introduction of corrections to account for factors such as propagation distance, site effects, and the discrepancy between the average velocity of the adopted crustal model, which is employed to generate the theoretical source spectra, and that of the reference site used for estimating site response.

Recently, the efficiency of the spectral amplitude at 3 Hz in capturing high-frequency radiation was confirmed from the analysis of a substantial data set^8^. As **m**_3Hz_ provides a more accurate representation of high-frequency ground motion variability than the currently employed magnitude scales in the entire range of magnitude of interest for seismic hazard assessment (i.e., M > 4), this could potentially result in a reduction in the scatter of ground motion intensity measures values in ground motion models. Indeed, the anticipated reduction in variability, which is currently regarded as random in ground motion models, has the potential to facilitate the development of more accurate seismic hazard models.

**Fig. 1:**
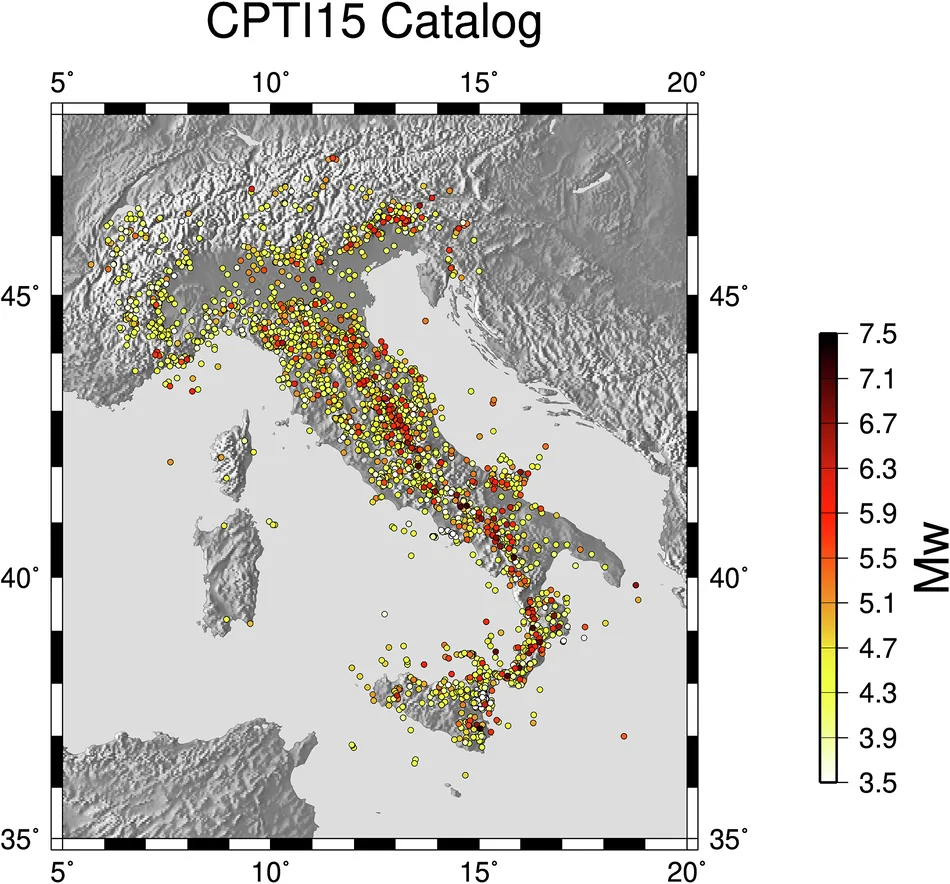
Map of earthquake epicenters from the CPTI15 Catalog. Only earthquakes with associated Mw are shown. Colors indicate the Mw magnitude of the events.

It was also demonstrated that the implementation of **m**_3Hz_ for the purpose of revising the estimates of the size of large earthquakes in historical catalogues is a conceivable prospect^7^. For this reason, we utilized empirical relationships that correlate macroseismic intensity with PGA and, in turn, with spectral amplitude at 3 Hz. We calculated **m**_3Hz_ for the events in the Parametric Catalog of Italian Earthquakes (CPTI15)^9,10^ (Fig. 1), for which macroseismic intensity data are available in the Italian Macroseismic Database (DBMI15)^11^. DBMI15 collects and harmonizes, after a quality check and validation procedure^12^, the macroseismic intensity data related to historical and recent earthquakes, as assessed by different kinds of investigations, from historical research to macroseismic field investigations of contemporary events. The current version of the database (DBMI15 version 4.0) contains, as a whole, 123,981 intensity data points referred to 20,162 localities and related to 3,229 earthquakes that occurred in Italy and surroundings in the period 1005 to 2020 CE.

After having implemented a selection of data based on a quality index (i.e., the variance-to-mean ratio, VMR), we present here the final **m**_3Hz_ catalog, which is denoted by the acronym ICEM (i.e., the **I**talian **C**atalog of **E**arthquakes based on the high-frequency earthquake **M**agnitude **m**_3Hz_) and comprises 2052 earthquakes (from 1117 to 2020), out of the 4894 in CPTI15.

## Method

The macroseismic intensity data for each event documented in DBMI15 was converted into PGA acceleration using invertible relationships^7,13^. The Intensity data were from DBMI15 v4.0; additional sources are listed in catalog files available on Zenodo^14^.

The PGA values were then utilized to calculate the spectral accelerations at 3 Hz, employing an empirical relationship that was calibrated with both the Japanese ground motion data set^7^ (2,858 records) for events with Mw between 5.7 and 6.9 (http://www.kyoshin.bosai.go.jp; last accessed for data download: August 2017), and a new dataset of 24,382 instrumental records concerning the Kahramanmaraş earthquake sequence of 2023^15^. For the Turkish dataset, strong-motion data (PGA, PGV and spectral amplitudes) were obtained from the data set described in Colavitti *et al*.^15^, which provides a comprehensive compilation of seismological observations for the East Anatolian Fault Zone (Türkiye). The processed accelerometric data used in this study are openly available on Zenodo^16^ at https://doi.org/10.5281/zenodo.13838992.

The analysis of these data facilitates the extension of the magnitude range of events to lower values (the data set for the Kahramanmaraş earthquake was collected for events with M_L_ magnitudes between 1.9 and 5.5), which allows for broad coverage of the minimum magnitudes (Mw 3.5–4) reported in the CPTI15 catalog. Furthermore, it enables the verification of variations in the relationship between PGA and spectral amplitude at 3 Hz in tectonic regimes that are completely different from one another. Please note that **m**_3Hz_ may not be suitable for estimating the magnitude of events with Mw less than 3.5–4, as it tends to underestimate it^7^.

**Fig. 2:**
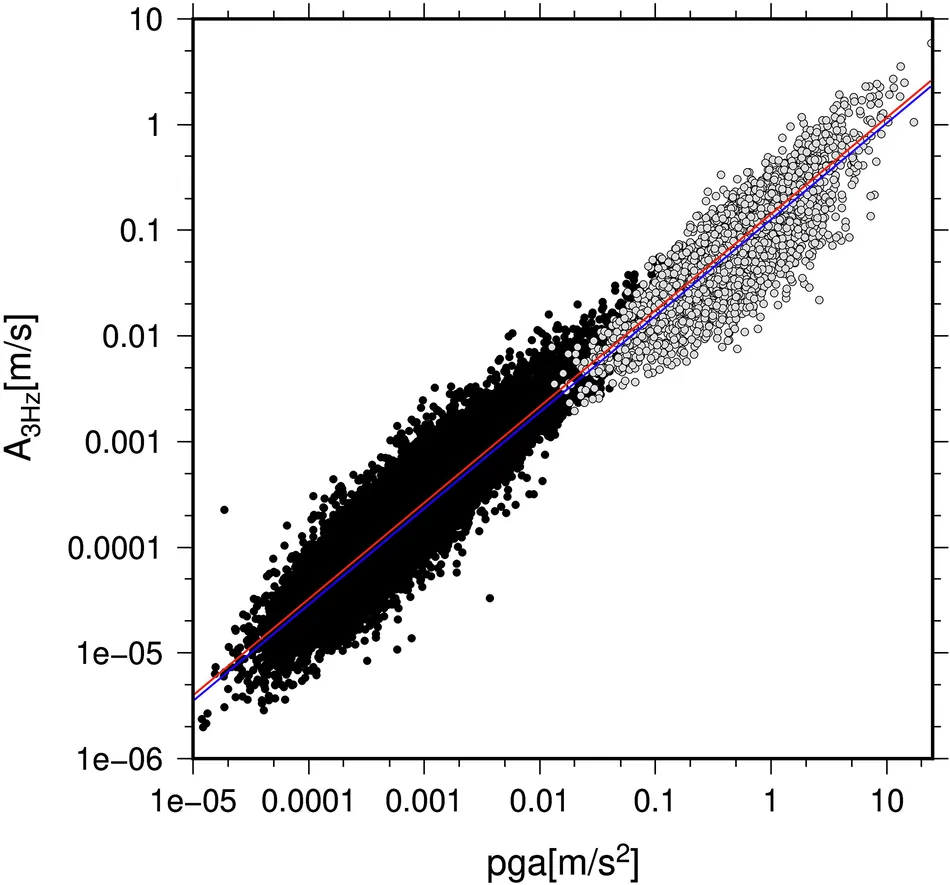
Spectral amplitudes at 3 Hz vs PGA obtained from the 27,240 horizontal component recordings of the Japanese (gray dots) and Kahramanmaraş (black dots) data sets. The red line indicates the regression of Eq. (1), while the blue line depicts the regression of Eq. (3) in Parolai *et al*.^7^.

As shown in Fig. 2, the datasets appear to be entirely complementary, in that one is simply an extension of the other, and there does not appear to be any significant bias in the linear trend, even for low PGA values which are likely associated with low-magnitude events for which the corner frequency is greater than 3 Hz.

Furthermore, similarly to previous work^7^, a relationship between PGA and spectral amplitude A at 3 Hz has been identified which, given the complementarity of the data discussed above, can therefore reasonably be used in other regions as well. This relationship, estimated despite the similarity of the data in order to take into account the new dataset that has become available, serves to eliminate any potential bias that may arise from the use of data originating from the same region as the tested area.

The relationship is in the form:

1 $$\log A=a\,log(PGA)+b,$$

where *a* = 0.910 ± 0.001 and *b* = −0.846 ± 0.004, *A* is in m/s and the *PGA* is m/s^2^.

The new relationship (red line in Fig. 2) is in excellent agreement with that previously calculated^7^ (blue line in Fig. 2), indicating that the relationship between PGA and spectral amplitude at 3 Hz is generally valid. It is noteworthy that alternative relationships, including polynomial ones, have been tested; however, these have resulted in only minor variations in the outcomes and are not presented. This equation was therefore used for the following analyses.

The **m**_3Hz_ magnitude was calculated for each site for which macroseismic intensity data are available (in the range 1 km to 200 km of epicentral distance) using the following formula:

2 $${{\bf{m}}}_{3{\rm{H}}{\rm{z}}}=2\log \left(\frac{A}{{A}_{{0}}}\right)+2\log \left(\frac{D}{{D}_{{0}}}\right)+5-{\Delta }_{site},$$

where *A* is the spectral amplitude of the acceleration measured at 3 Hz (in m/s), and *A*_*0*_ is the reference spectral amplitude at 3 Hz of the source function of the single corner frequency model at 10 km, obtained by considering an M_W_ = 5 event and assuming a stress drop of 1 MPa. According to Parolai *et al*.^7^, an ω^−2^ model^17,18^ was assumed, and the shear wave velocity was set to 3500 m/s, resulting in an *A*_*0*_ value of 0.029525 m/s. In the second term of Eq. (2), D is the epicentral distance (km) from the CPTI15 (i.e., we consider the epicentral distance because the depth of historical events is not assessed) and *D*_*0*_ is the reference distance which is set to 10 km.

It should be noted that using the epicentral distance instead of the hypocentral distance can lead to an underestimation of the magnitude for the macroseismic intensity values observed closer to the hypocenter. This underestimation obviously becomes less significant as the epicentral distance increases. However, considering that 70% of the events in the area have an estimated depth of less than 15 km, and 50% less than 10 km, and further considering that the epicentral distances in the dataset exceed 15 km in 75% of cases, it is reasonable to expect that this underestimation effect occurs in only a few cases and that this effect is smoothed out in the event magnitude estimate that uses numerous observations.

${\varDelta }_{{site}}$ is the site amplification factor, estimated using the V_s30_ value, the time-averaged shear-wave velocity (Vs) to a depth of 30 meters^19^ which is available at each (or nearby to each) macroseismic intensity data point location, through a map^20^ by:

3 $${\varDelta }_{{site}}=2{\log }{\left(\frac{{V}_{s30}}{{Vm}}\right)}^{1/2}$$

**Fig. 3:**
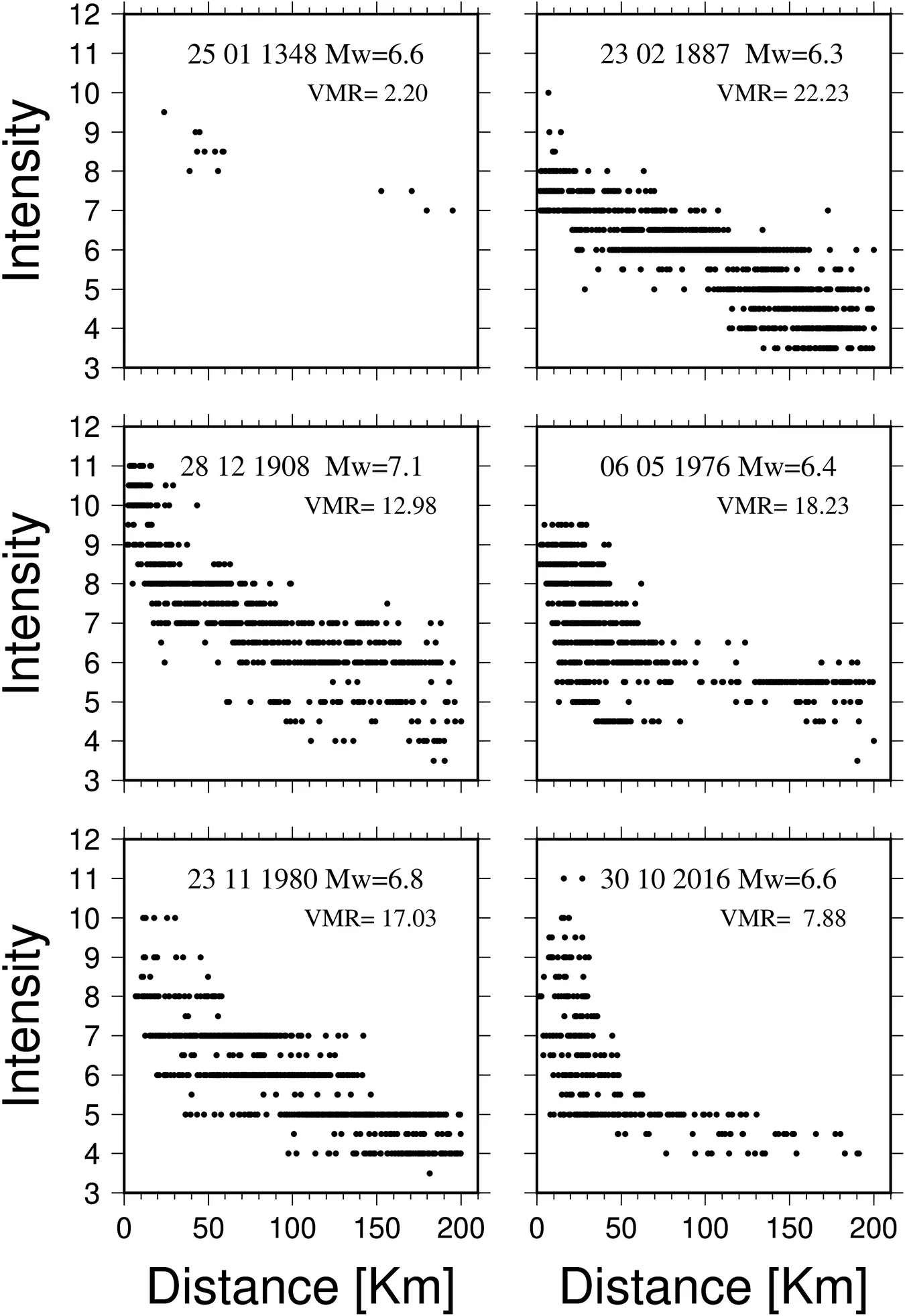
Macroseismic intensities versus epicentral distances (selected in the range 10 to 200 km) for six significant earthquakes that occurred in Italy: the 1348 Friuli earthquake (top, left), the 1887 Liguria earthquake (top, right), the 1908 Messina earthquake (middle, left), the 1976 Friuli earthquake (middle, right), the 1980 Irpinia earthquake (bottom left) and the 2016 Norcia earthquake. Each panel displays the VMR for each event.

Δ_*site*_ accounts for the difference in impedance between the velocity in the shallow geology represented by V_s30_ and the adopted velocity model (*Vm*), thereby combining the ${\varDelta }_{{site}}$ and the ${\varDelta }_{{{site}}_{{ref}}}$ terms^7^. Note that, unlike for the application to strong motion data as previously proposed^7^, a correction factor to account for a possible path-dependence of anelastic attenuation correction from magnitude estimates at 10 km distance was not necessary, probably due to the high dispersion of the Intensity data points with respect to distance (Fig. 3).

Data from DBMI15 with intensities not expressed as numerical values (e.g., “F” for felt, “HD” for heavy damage^9,11^) were discarded. A total of 69,734 intensity data related to 2,415 earthquakes was processed.

**Fig. 4:**
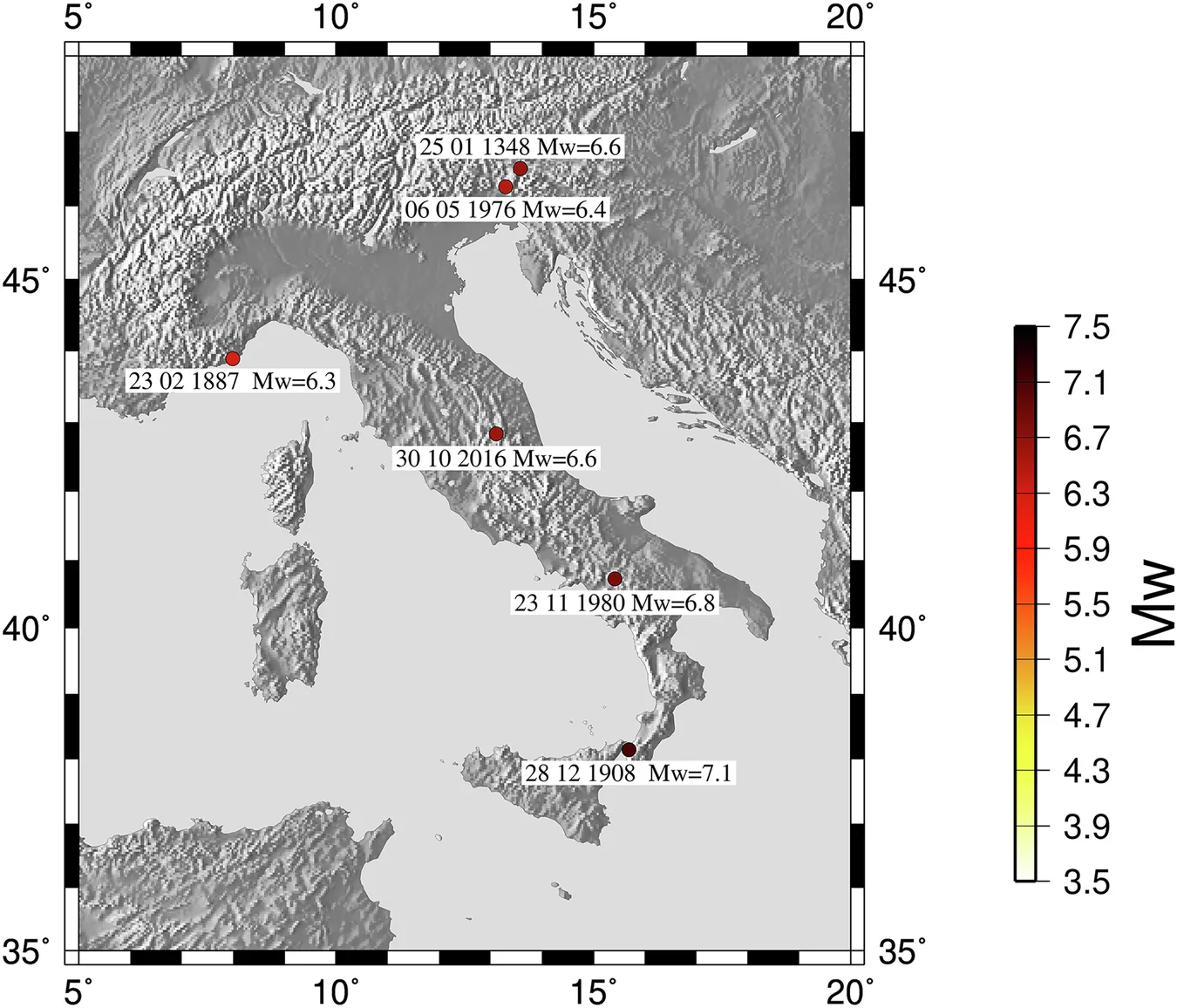
Epicentral positions of the earthquakes shown in Fig. 3: the 1348 Friuli earthquake, the 1887 Liguria earthquake, the 1908 Messina earthquake, the 1976 earthquake, the 1980 Irpinia earthquake and the 2016 Norcia earthquake.

In Fig. 3, the macroseismic data employed for certain events of particular significance (and covering various regions of Italy) is presented. It is evident that data scattering becomes significant for low intensity values, and the tendency for macroseismic intensity to decrease with increasing distance depends on the location (region) of the event (Fig. 4). Furthermore, the figure shows how some past events (e.g., that of 1348) may suffer from a lack of reliable intensity data.

The final magnitude of the event is calculated using a trimmed mean, in which magnitude values that deviate from the mean value by +/− one standard deviation are discarded. The final standard deviation is calculated using only the selected values.

Furthermore, for each event (and therefore for each **m**_3Hz_ estimate), a quality index was computed to quantify the dispersion of macroseismic intensity data as a function of distance, with particular emphasis on the capability to describe its attenuation trend. Experimental tests showed that the most effective parameter for representing this capacity is the variance-to-mean ratio (VMR)^21^, which we use as a proxy for data quality. To compute the VMR, the distance–intensity domain of each event is discretized into cells, obtained by partitioning the horizontal axis into 10-km distance intervals and the vertical axis into 1-intensity-unit bins.

The VMR is then calculated as:

4 $${VMR}=\frac{{\sigma }^{2}}{\mu }$$

where *μ* is the average of the values counted across all cells and *σ*^2^ is the variance between cells.

In the case in question, high VMR values (>1) indicate an oriented density and a structured pattern, while values around 1 indicate a random distribution of data. Consequently, a high value is deemed appropriate for the description of a trend curve. This phenomenon can be attributed to the presence of a significant number of empty cells in which the distance-intensity plane has been divided, in conjunction with other cells exhibiting large numbers of data points. This results in a high variance from the mean, consequently leading to a high VMR. In conclusion, VMR is effective in this context because it reflects clear variations in data density between different areas of the plane and rewards the consistent concentration of points, which is precisely what makes a curve representative. It also exhibits a natural threshold (VMR approximately equal to 1) that distinctly differentiates uniformity and structure.

## Data Records

The datasets are available at https://doi.org/10.5281/zenodo.20232883^14^.

Note that this study releases two datasets:

The ICEM (ICEM.txt) catalog, provided in plain-text format and hosted by Zenodo which is a free, open-access digital repository platform developed by CERN (European Organization for Nuclear Research) in collaboration with the European Commission and is now part of the open-research infrastructure supported by the EU at https://doi.org/10.5281/zenodo.20232883. This catalog contains the 1,951 earthquakes for which the estimated VMR is greater than 1, covering the period 1117–2020.

The ICEM “review catalog” (ICEM_review_catalog.txt), available at the same website https://doi.org/10.5281/zenodo.20232883.

This dataset includes events with VMR > 1 that nevertheless require particular caution in their use because (i) the magnitude estimates rely on a small number of macroseismic intensity data, or (ii) the difference between **m**₃_Hz_ and Mw exceeds one magnitude unit. Most of these events occur in volcanic areas or outside Italy.

For all other events, the use of the data is recommended provided that the associated VMR value is properly taken into account.

Both catalogs share the same data structure.

Each record reports the following fields, in order of appearance:

EV – unique event identifier as defined in DBMI15/CPTI15;

Ind – event index in the CPTI15 catalog;

LatEV – event latitude (from CPTI15);

LonEV – event longitude (from CPTI15);

DepthEV – focal depth (in km) when available (from CPTI15; 99 denotes missing values);

MwM – Mw estimated from macroseismic intensity (CPTI15);

Mwdef – default Mw reported in CPTI15;

MWIn – instrumental Mw when available (CPTI15);

M3hzEV – **m**₃_Hz_ magnitude estimated in this study;

stdvEV – standard deviation of **m**₃_Hz_;

ndat – number of macroseismic data points used in the **m**₃_Hz_ computation;

VMR – variance-to-mean ratio estimated for the event;

Var – variance used in the VMR calculation;

vmed – mean value used in the VMR calculation;

Reference – Event reference;

CODE – bibliographic reference code;

DOI – Digital Object Identifier for the referenced publication or dataset;

URL – Direct link to the publication, dataset. These files are distributed together with a README.md (https://doi.org/10.5281/zenodo.20232883) describing the structure of the datasets and recommendations for their use.

### Data overview

In order to encourage the utilization of the ICEM Catalog, a list of its principal features is provided below.

**Fig. 5:**
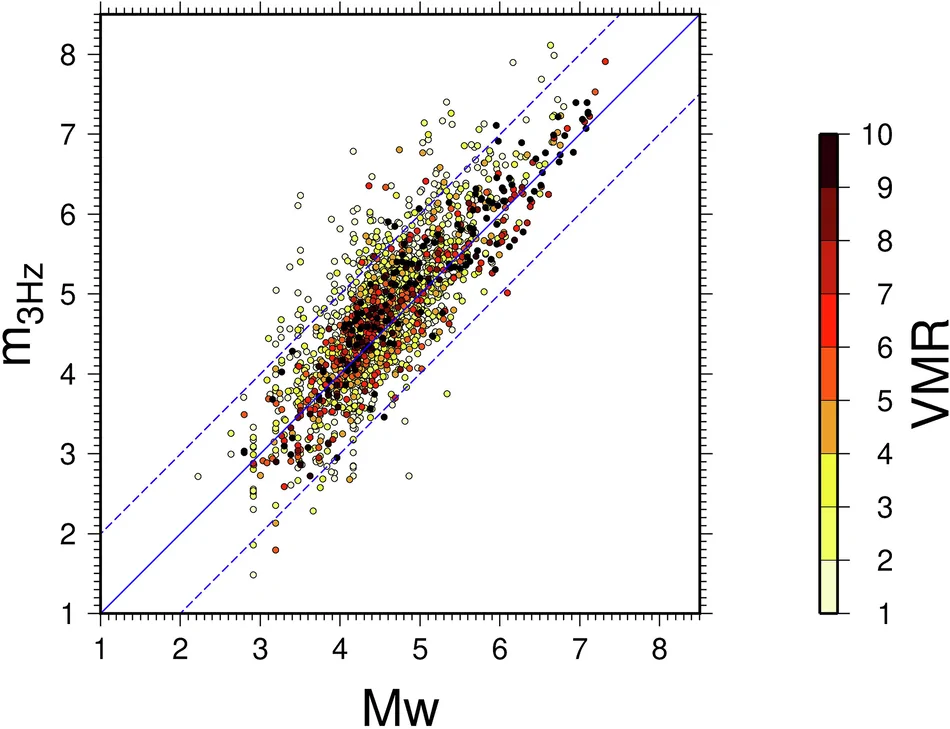
The data set presented in the catalogue is displayed as a function of **m**_3Hz_ versus Mw. The color of each event indicates its VMR value. The blue line indicates the 1:1 ratio. The dashed blue lines indicate the +1 and −1 magnitude units.

Figure 5 presents a comparison between the Mw assigned in the CPTI15 catalogue to the selected events and their **m**_3Hz_ estimated in this study.

The color of each event indicates its VMR value. Overall, the data show a linear relationship. As discussed above, the dispersion of the data is due to the different ways in which the two magnitude scales look at the seismic source (i.e., Mw considers the average static displacement of the rupture and its slip area; **m**_3Hz_ considers the amount of energy radiated in the high frequencies). Furthermore, **m**_3Hz_ is estimated by considering a reference value as defined in Eq. (2). However, it should be noted that, in general, the greatest differences occur for lower VMRs. A trend toward lower VMR values is observed for events with absolute differences between **m**_3Hz_ and Mw greater than 1. However, these events are the ones previously identified as requiring caution. Occasionally, and in accordance with the reasons outlined in the Method section, events with a magnitude of approximately Mw 3 have low **m**_3Hz_ magnitudes (up to 1.5). Below, we will further discuss the difference between Mw and **m**_3Hz_.

**Fig. 6:**
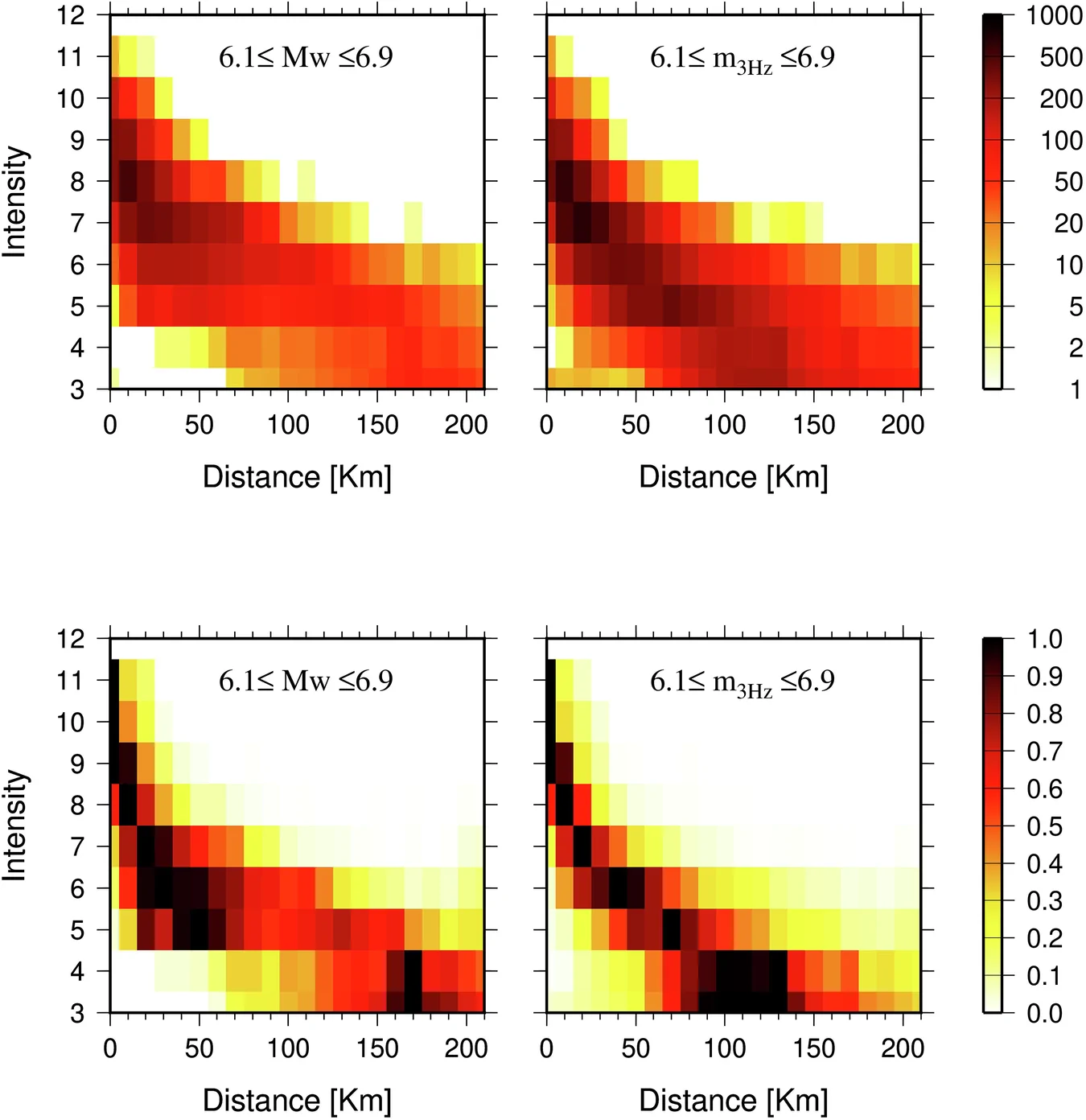
The graph shows the number of data points as a function of distance and macroseismic intensity value (top panels). The data selected was based on Mw ranging from 6.1 to 6.9 (left). The data selected was based on the **m**_3Hz_ range of 6.1 to 6.9 (right). The number of data points that have been normalized based on the maximum number of data points for each intensity grade, as a function of distance and macroseismic intensity value (bottom panels). The data selected considering Mw spanning between 6.1 and 6.9 is shown on the left. The data selected considering **m**_3Hz_ between 6.1 and 6.9 is shown on the right.

An important aspect of the ICEM catalog can be seen in Fig. 6, which shows, as an example, the distribution of the number of macroseismic intensity data versus epicentral distance for events with Mw (Fig. 6, top left) and **m**_3Hz_ (Fig. 6, top right) between 6.1 and 6.9. The two panels show how a higher number of data tend to cluster better in a curve indicating a decrease in macroseismic intensity with distance when the selection is made on the basis of the **m**_3Hz_ associated with the events in this catalog. This is even more evident in the lower panels of Fig. 6, which show the values from the previous panels normalized to the maximum value for each degree of macroseismic intensity. Since the number of macroseismic intensity data points varies depending on both the macroseismic intensity itself and the distance, normalization allows for a clearer visualization of the clustering of points (for a given macroseismic intensity) at certain distances. The decrease in the spread of data is evident when **m**_3Hz_ is used. This confirms that the use of **m**_3Hz_ can lead to a reduction in the scatter of ground motion intensity measurement values in ground motion models, thus reducing part of the uncertainty that is currently considered random.

**Fig. 7:**
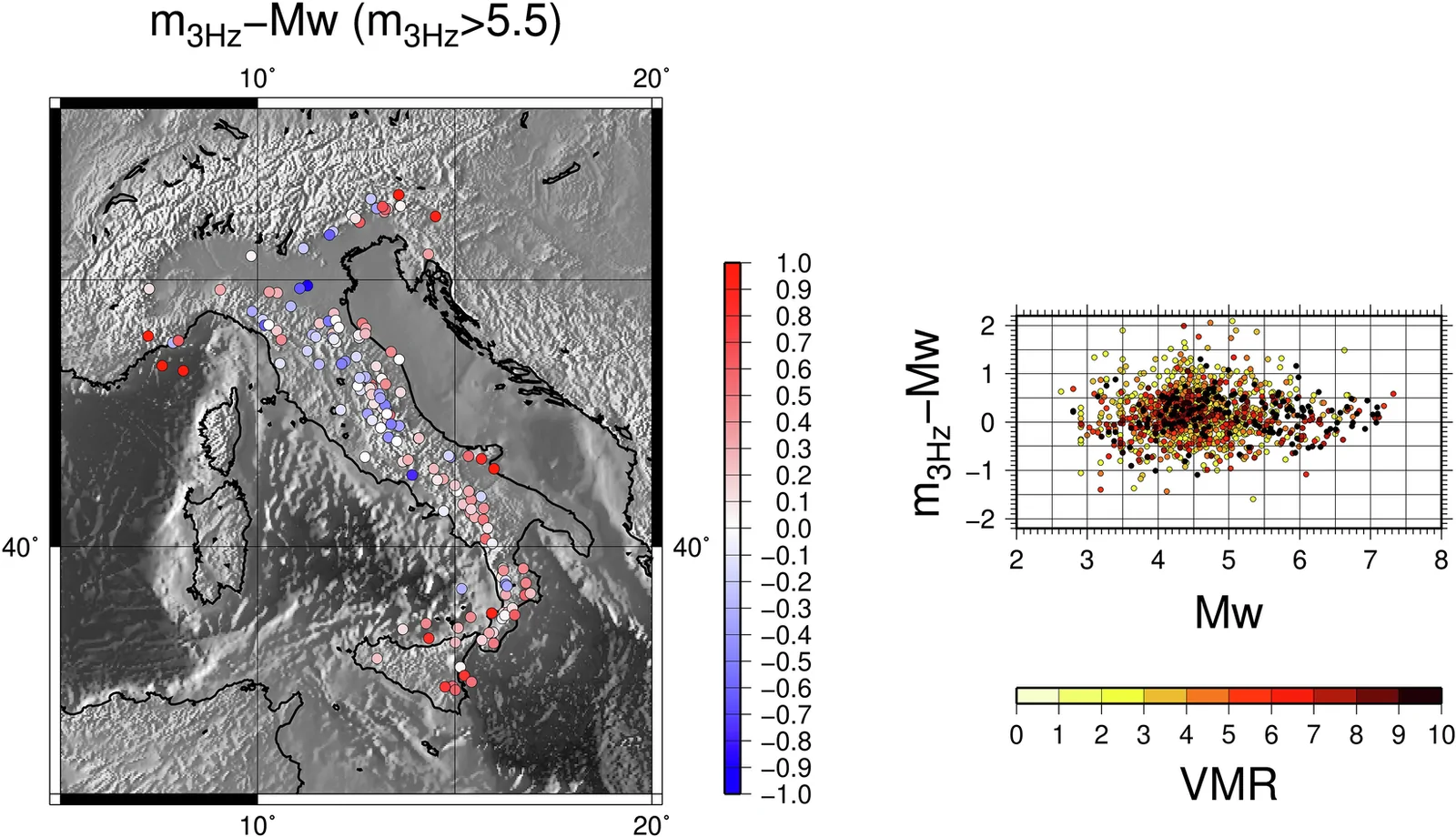
Left: The map shows the differences between the **m**_3Hz_ and Mw values of earthquakes with **m**_3Hz_ greater than 5.5 in the ICEM catalog. There are clear patterns in the positive (larger high-frequency content of the shaking) and negative (smaller high-frequency content of the shaking) values: positive values in the Ligurian Sea and the Alps; negative values in the northern and central Apennines; positive values in the Adriatic foreland and the Gargano; and neutral to positive values in the southern Apennines, Calabria, and eastern Sicily. Right: the differences between the **m**_3Hz_ and Mw versus Mw. The color of each event indicates its VMR value.

Coming back to the difference between the **m**_3Hz_ and Mw values, ΔM, Fig. 7 shows as the spatial distribution of ΔM follows a clear pattern, with higher values in the northeastern Alps and southern Apennines, while lower values are clustered in the central Apennines. These variations, which indicate variability in high-frequency shaking at the same Mw, are due to the combined effect of seismic wave attenuation and variations in high-frequency source radiation. They have a clear regional connotation, reminiscent of the tectonic zoning^22^. It should be noted that these correspond well to attenuation variations observed in previous instrumental data studies^23–25^ and macroseismic intensity data studies^26^, as well as to seismic wave velocity distributions at the crustal level throughout Italy^27,28^.

The right panel of Fig. 7 shows the differences between **m**_3Hz_ and Mw as a function of Mw for all events in the catalog. Within the range analyzed in this study, no systematic variations dependent on Mw are observed, although the scattering seems to be larger for events with Mw smaller than 5.5.

It is difficult to compare the results of this study with those of a recent study^29^ that used a single attenuation model for most of the territory considered in the catalogue. However, it is important to note that they emphasize the importance of regionalizing attenuation models. Regarding the variability of the stress drop^29^, depending on the attenuation models used, the results of this study suggest that, within the range of epicentral distances considered for **m**_3Hz_ estimation, attenuation could predominate over high-frequency radiation alone at the source.

## Technical Validation

It is of fundamental importance to assess the possible application of **m**_3Hz_ to macroseismic intensity data for its use in seismic hazard assessment procedures. In this regard, six events listed in the ICEM catalog that occurred in Central Italy were examined, for which a significant number of strong-motion records are available. For these events, **m**_3Hz_ was also estimated using the available strong-motion data^7^. The recordings relate to the mainshock of the 2009 earthquake in L’Aquila and the 2013 earthquake in Matese. They also relate to four events of the Central Italy sequence, which occurred on 24 August and 26 and 30 October 2016 and 18 January 2017.

For this Italian accelerometric dataset, records and parametric information were retrieved from the Observatories and Research Facilities for European Seismology (ORFEUS) European Integrated Data Archive (https://www.orfeus-eu.org/data/eida), the Incorporated Research Institutions for Seismology (IRIS) (https://www.iris.edu/hq/), and the Italian Civil Protection Department (http://ran.protezionecivile.it/EN/index.php). The National Institute for Geophysics and Volcanology (INGV) bulletin is used to guide the data download (webservices.rm.ingv.it/fdsnws/event/1/) and to extract the earthquake parameters (last accessed September 2020). The International Federation of Digital Seismograph Networks specifications are available at http://www.fdsn.org/ and the Standard for the Exchange of Earthquake Data manual is available at http://www.fdsn.org/pdf/ SEEDManual_V2.4.pdf. The seismic recordings utilized in this study primarily originate from the following seismic monitoring networks: INGV, (https://doi.org/10.13127/SD/X0FXnH7QfY), Presidency of Council Of Ministers-Civil Protection Department (https://doi.org/10.7914/SN/IT), MedNet Project Partner Institutions, https://doi.org/10.13127/SD/fBBBtDtd6q), University Of Bari “Aldo Moro” (https://doi.org/10.7914/SN/OT), Amatrice Sequence International (https://doi.org/10.7914/SN/YR_2016), Rete sismica del gruppo EMERSITO (https://doi.org/10.13127/SD/7TXeGdo5X8), Seismic network RESIF-SISMOB (https://doi.org/10.15778/RESIF.XJ2009) and 3 A (Rete del Centro di Microzonazione Sismica (CentroMZ), (https://doi.org/10.13127/SD/ku7Xm12Yy9).

**Fig. 8:**
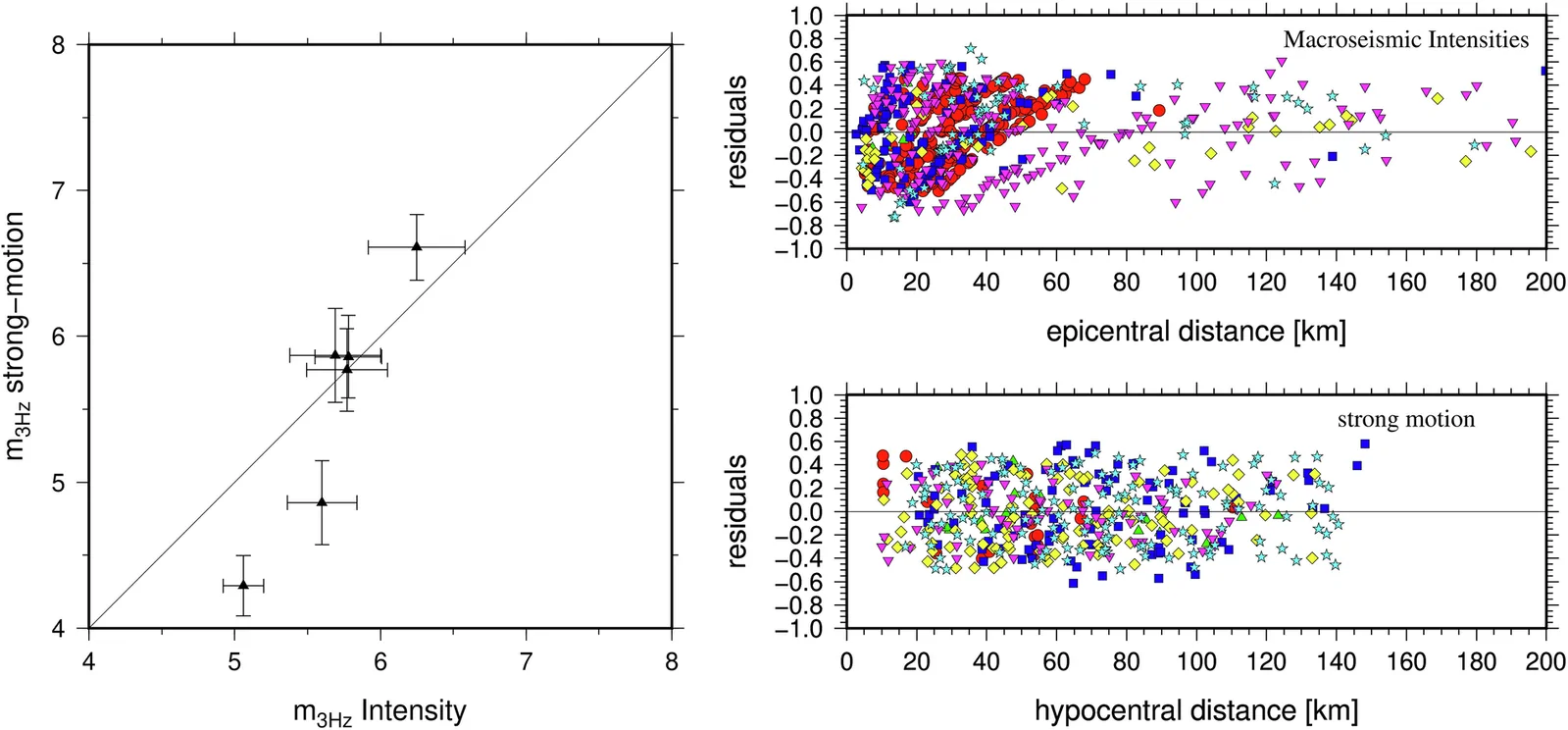
Left) A comparison is made between the **m**_3Hz_ values calculated using strong motion recordings and macroseismic intensity data for six events: the 2009 L’Aquila earthquake; the 2013 Matese earthquake; and four events in the Central Italy sequence which occurred on 24 August and 26 and 30 October 2016, as well as on 18 January 2017. Note how the scales on the x- and y-axes have been zoomed in compared to Fig. 5. Right) Magnitude residuals versus hypocentral distance for the 2009 L’Aquila earthquake (red circles), the 2013 Matese earthquake (green triangles), the 24 August (blue squares) and 26 (yellow diamond) and 30 October (purple inverted triangle) 2016 Central Italy events, the 18 January (light stars). The bottom panel displays the residuals obtained by calculating **m**_3Hz_ with strong motion data. The top panel presents the residuals obtained by using macroseismic intensities.

The values of **m**_3Hz_ obtained from the macroseismic intensity data (Fig. 8) are consistent with those estimated from the strong-motion data. In two cases, the **m**_3Hz_ value derived from strong-motion data is lower than that derived from macroseismic intensity; however, the latter is closer to the Mw value reported in ICEM. The two methods exhibit similar levels of estimation precision (0.2–0.3) and the same trend in defining relative event sizes. The precision of the estimations is better than that in Parolai *et al*.^7^ because this study considered site effects and used a trimmed mean. Obviously, the precision of the **m**_3Hz_ estimates from macroseismic data could be lower if the propagation of uncertainties in the conversion relationships between macroseismic intensity and PGA, and between PGA and spectral amplitude at 3 Hz, were taken into account. Although this analysis will be the subject of future studies specifically dedicated to this topic and which will benefit from the data set provided in this work, the fair agreement between the average estimates obtained from recordings and those from macroseismic data remains remarkable.

Figure 8 also shows the distribution of station residuals with respect to the average value of the estimated magnitude. In general, the residuals do not show any trend with magnitude. Only for the 2009 L’Aquila event, when the **m**_3Hz_ is estimated with strong motion data, can a certain trend be observed in the first 20–30 km, but the scarcity of data does not allow us to go further in this interpretation. A certain observable trend in the residuals (in the case of macroseismic data) to increase with distance—but only for certain limited distance ranges and only for a couple of events—is due to the distance correction combined with the fact that macroseismic intensities remain constant over certain distance intervals. However, the fact that no trends in the residuals are observed over the total distance range considered demonstrates the appropriateness of the correction performed.

**Fig. 9:**
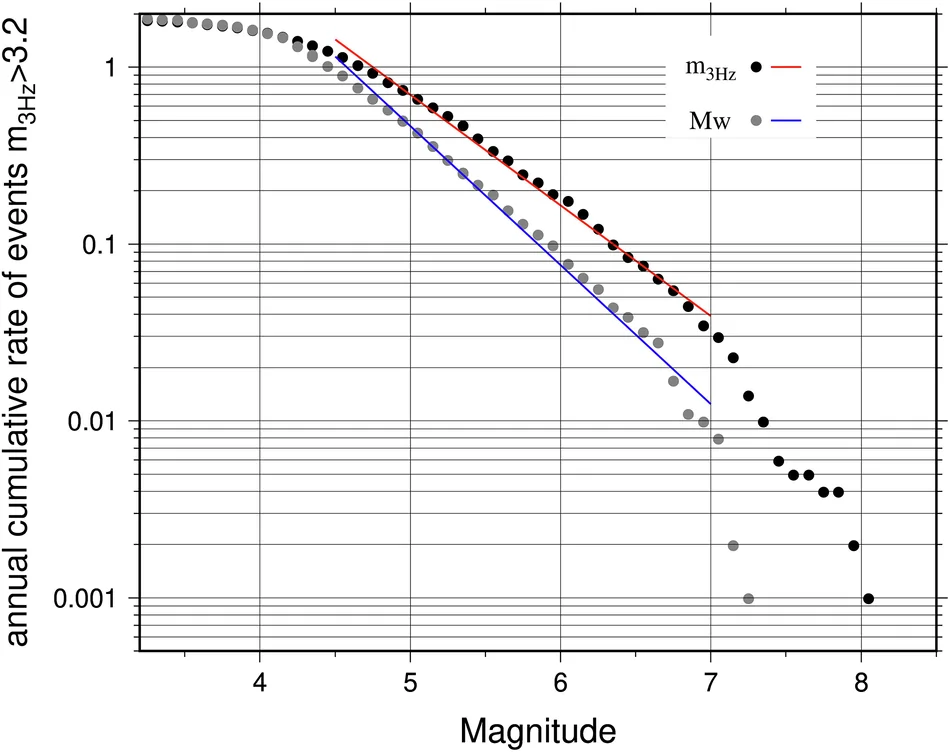
The annual cumulative rate is calculated using **m**_3Hz_ (black dots) and Mw (grey dots). The best-fit lines are depicted within the regression range: red for **m**_3Hz_ and blue for Mw.

Aiming at highlighting how different event size metrics (i.e., Mw and **m**_3Hz_) can affect earthquake statistical outcomes, we show in Fig. 9 the b-values estimated on the ICEM catalog. The b-value was calculated for events with magnitudes between 4.5 and 7, in magnitude bins of width 0.2 and with a shift of 0.1.

To calculate the annual cumulative rate, events with a magnitude greater than 3.2 were used, which on a national scale may also include some volcanic events. Since the purpose of the study is not to calculate the b value for seismic hazard studies, we have not made any distinctions between events, nor have we made any regional distinctions. However, we emphasize this aspect so that those who wish to use the catalog for further studies in the future are aware of its limitations and the need for careful analysis of the data it contains. Nevertheless, the estimated b values provide a clear first-order picture of the impact of using different magnitude scales in calculating the b value.

In fact, the b-value equal to 0.8 obtained for Mw is consistent with a reported estimate^30^, who obtained a value of 0.74 for Central Italy. The b-value obtained using **m**_3Hz_ is 0.62. Differences between b-value estimates by different metrics (i.e., magnitude scale) have been shown to connect to variations in stress^5^. We therefore foresee that b-value estimates retrieved from the integrated analysis of Mw and **m**_3Hz_ catalogs developed for seismic hazard assessment will provide useful constraints.

## Data Availability

The dataset, the ICEM catalog, is publicly available at Zenodo (https://doi.org/10.5281/zenodo.20232883). The repository contains the catalogue files (ICEM.txt and ICEM_review_catalog.txt) and a README.md file describing its structure and use.

## Code availability

No custom code has been developed to extract information from the ICEM catalog, which can be analyzed using code developed in any standard language. The *Generic Mapping Tools* (www.soest.hawaii.edu/gmt)^31^ were used for creating the Figures.
